# Bringing Molecular Tools into Environmental Resource Management: Untangling the Molecules to Policy Pathway

**DOI:** 10.1371/journal.pbio.1000069

**Published:** 2009-03-31

**Authors:** Raphael Sagarin, Jens Carlsson, Michelle Duval, Wilson Freshwater, Matthew H Godfrey, Wayne Litaker, Roldan Muñoz, Rachel Noble, Tom Schultz, Bennett Wynne

## Abstract

New advances in molecular biology can be invaluable tools in resource management, but they are best incorporated through a collaborative process with managers who understand the most pressing questions, practical limitations, and political constraints.

Increasingly, scientists are drawn to public debates on environmental policy, yet find themselves ill-equipped to influence the outcome. While many scientists have collected data (for example, on species being considered for listing under the Endangered Species Act) or developed technologies (for example, to detect unregulated waterborne pollutants) relevant to current policy debates, communicating these results to policy makers is no guarantee that a rational policy response will follow. Biologists continually overemphasize the technical aspects of their work and almost completely ignore the social-political environment in which their work is meant to inform. Specifically, most biologists seem to believe that if they work out the technical hurdles and then effectively communicate their science to policy makers, their work will affect and change policy. This is a grievous mistake and one that has continued to reinforce the science/policy divide, rather than anneal it. Scientists who do receive training (RS was 2002–2003 Congressional Science Fellow of the American Association for the Advancement of Science, under the sponsorship of the Geological Society of America) quickly learn about the “three Ps”—policy, politics, and process—that govern lawmaking. Scientists tend to focus overwhelmingly on the first “P,” because policy is the one area where data and scientific expertise may be brought to bear. But policy does not move forward without attention to the often complex politics behind the policy, or the bureaucratic processes that must be navigated. Even once policy is made, its implementation may not follow the most scientifically appropriate methods. This is both because improved techniques may have been developed after the policy was enacted and because managers constrained by legislatively mandated protocols (no matter how outdated) have limited opportunity for feedback to policy makers.

We believe that scientists and resource managers charged with implementing environmental policy can develop meaningful dialogues to navigate and in some cases streamline this complex “science to policy” pathway. This pathway can be redrawn as an iterative, collaborative approach in which researchers and managers discuss current and anticipated resource management needs and questions, researchers describe how scientific tools can meet those needs, and managers articulate the political and bureaucratic challenges that must be overcome to incorporate those tools. Opening a dialogue between managers and molecular researchers catalyzes feedback on policy and management by identifying: (A) outstanding questions that cannot be answered with currently approved protocols; (B) places where existing policy requires the use of potentially misleading “indicators” (e.g., measuring chlorophyll a as a surrogate for nitrogen/eutrophication, measuring fecal indicator bacteria for water quality testing rather than specific human viral and bacterial pathogens); and (C) inefficiencies in existing protocols.

As a model for this pathway, we focus on molecular biology because it presents new techniques that might improve management of environmental resources. However, adopting them would require a wide range of changes in management protocols and policy, as well as data interpretation and database management. While there are technical hurdles in adapting new lab techniques for field management situations, molecular techniques are already being employed in environmental management, and we argue that the bigger challenge to implementation is in surmounting the political, bureaucratic, and process-oriented hurdles. Here we outline some cutting-edge examples from seafood monitoring and fisheries management to show the promise of molecular approaches to management, and then discuss water quality monitoring as an example where promising new molecular techniques are running into institutional barriers on their way to implementation. We conclude with an illustrative example of a “Molecules to Policy” dialogue we created in the southeastern United States that provides general lessons for navigating this complex pathway.

## Applying Molecular Techniques to Resource Management

Certainly, for management agencies with tight budgets the cost and complexity of molecular techniques is a primary concern. Nonetheless, the cost of molecular techniques is rapidly decreasing [1], and protocols are increasingly being packaged into ready-made, user-friendly kits. Alternately, agencies can outsource molecular tests on a fee-for-service basis or develop partnerships with researchers through the type of collaborative working groups described below. Clearly, molecular approaches must be tested under real world circumstances and should be compared with already approved methods using side by side testing of “traditional” versus new molecular methods. Indeed, combining molecular approaches with traditional approaches is a vital step, as the redundant measures can help cross-check the accuracy of results, reveal the relative merits of each approach, and highlight synergies where combined approaches provide considerably more information than either approach alone. The following examples illustrate successful recent deployments of molecular techniques in management situations.

### Molecular techniques for evaluating protocols.

Numerous factors, ranging from overfishing to pollution and disease, have resulted in the collapse of the eastern oyster (Crassostrea virginica) fishery in Chesapeake Bay. Restoration strategies have included limits on commercial and recreational harvest, reef restoration, oyster translocation, and supplementation of existing wild populations through deployment of hatchery-reared oysters. It has been hypothesized that crossing hatchery strains selected for disease resistance and growth might retain their selected advantages while avoiding the effects of inbreeding common in domesticated stocks, making these cross-breeds particularly useful in restoration efforts. Cordes et al. [2] utilized data from molecular markers called microsatellites to discriminate among all hatchery lines deployed in the field, and determined that counter to the goals of the restoration policy, the hatcheries did not effectively produce hybrids prior to release. This study highlights the importance of genetic tools for retrospectively monitoring restoration efforts, and suggests that they could also be used prior to deployment of oyster seedlings to ensure quality control and avoid wasting resources.

### Molecular techniques in real time management.

Differentiation of stocks is essential to fisheries management—especially in species with strong geographic affinity to particular spawning streams such as Pacific salmon—to ensure the maintenance of genetic diversity and preservation of robust spawning stocks spread across the geographic range. West Coast fisheries managers initially explored several different genetic markers and techniques to identify stocks, but due to technical difficulties, lack of resolution (i.e., where genetic variability is small, it is difficult to detect genetic differences), or low throughput (i.e., labor-intensive work that limits the number of individuals that can be processed), discarded most markers (e.g., restriction fragment length polymorphism, amplification fragment length polymorphism, allozymes, and DNA sequencing). Microsatellites meet the high-throughput (multiple microsatellites can be handled simultaneously) and resolution (large amount of genetic variability) criteria, yet comparing data from different laboratories is problematic because the inferred length variation (measured in base pairs) could differ significantly depending on the equipment used [3]. While it is possible to standardize microsatellite typing among labs (as federal agencies have done for crime forensics), it is an expensive and laborious process that is poorly adapted to implementation and advances of new technology. Thus, West Coast fisheries managers settled on single nucleotide polymorphism (SNP) markers, which have made great improvements in accuracy and throughput recently [3] at reduced expense. Alaska Department of Fish and Game managers in the Bristol Bay can now use SNP data from 2,000 fish taken in a test fishery, compare them to a baseline of 10,000 fish, and assign them to a source population, allowing managers to estimate stock compositions and set stock-specific fishing targets within 24 hours [4]. Without this up-to-date information, particular stocks, which are superficially identical and may mix in open water, could be disproportionately overexploited, leading to the collapse of geographically isolated spawning streams. Genetic stock identification also allows for proactive management in which stocks that are known to have declining spawning populations can be protected from overfishing.

## Challenges Illustrated by Water Quality Testing

Despite real world successes, challenges incorporating molecular techniques into management remain. Though some hurdles are purely technical, it is the “process” challenges—related to how new techniques are approved, incorporated into the required/recommended protocols by different agencies, and implemented on the ground—that may prove to be most difficult. Many of these challenges are illustrated in the cases of water quality testing for edible seafood and recreation, where new technologies could vastly improve accuracy, precision, and timeliness of management.

New rapid quantitative polymerase chain reaction (QPCR) assays can rapidly quantify fecal indicator bacteria in recreational waters [5] and quantify the likely health risks to swimmers because the results have been shown to be directly related to human health outcomes [5–7]. These tests, which have been favorably compared to existing US Environmental Protection Agency (EPA)–approved methods such as membrane filtration and substrate kits [8], yield results in less than two hours, compared to 18–96 hours for culture-based tests. For a typical water quality laboratory, the new techniques require an initial purchase of QPCR equipment, but the individual rapid QPCR water quality tests can be conducted at a cost that is the same or only slightly greater than that for currently utilized tests. Sample processing can occur at roughly the same overall throughput as existing water quality assays. For example, a typical QPCR machine can process 96 samples simultaneously, and a technician can process roughly 24 samples at a given time; these numbers are similar to or exceed the processing possible by currently used culture-based tests. Several hurdles would need to be overcome before molecular tests could be widely implemented, including research to assess the persistence of DNA (the measured QPCR endpoint) as compared to metabolically active bacterial cells (the measured endpoint of membrane filtration and defined substrate technology approaches). It is also necessary to determine how to integrate data from the currently used culture-based tests and new rapid molecular methods in long-term monitoring efforts such as total maximum daily load (the total amount of a pollutant allowable in a given water body) development and implementation. Finally, managers and technicians used to culture-based methods also need to be trained in the theory behind molecular methods, giving them the ability to perform the tests reproducibly and with confidence in the results.

Longstanding divergence in agency mandates—in some cases, arising from different economic and risk-analysis considerations—as well as historical precedent within the regulatory agency can complicate efforts to adopt molecular approaches. An agency that routinely deals with high levels of uncertainty but does not directly concern itself with human health or public health risk (as in fisheries stock assessment) is likely to be much more amenable to quickly incorporating new molecular techniques than agencies that deal with water quality (recreational or seafood), which themselves will diverge based on the potential consequences for human health. For example, recreational water quality managers in Southern California, where beach use represents a US$3.5 billion industry [9], generally prefer an approach that is rapid but minimizes type I error (posting an advisory when no danger exists), whereas a North Carolina shellfish water quality manager generally will want to minimize type II errors (failure to post when a real risk is present) because the potentially lethal health consequences of shellfish poisoning presumably outweigh the cost to the US$32 million shellfish industry [10].

In North Carolina, recreational water quality managers work out of the same state water quality agency as the shellfish water quality managers (Department of Environmental and Natural Resources), yet each entity answers to a different agency (EPA or Interstate Shellfish Sanitation Conference [ISSC]), with vastly different procedures for approving new protocols. The ISSC approval process requires specific procedures for different tasks, whereas the EPA recommends a protocol based on a range of available “EPA-approved” methods, allowing recreational water quality managers to pick and choose which methods fit in best with their needs, resources, and personnel capabilities. Getting new methods approved by the EPA and ISSC, then, requires different pathways for methodological validation and acceptance.

To introduce new methods for monitoring shellfish water quality, managers must endure a painstaking process that typically can take several years to complete. Protocols for managing water quality for shellfish in the US are rooted in the National Shellfish Sanitation Program, first developed in 1925 in response to typhoid fever outbreaks associated with contaminated shellfish. These testing protocols are tightly controlled by the ISSC, standardized for all coastal states, and must be conducted in certified facilities. In most places in the world, managers base decisions about shellfish safety on assays of Escherichia coli densities, which could be straightforward to test using QPCR methods. Shellfish managers outside the US are considering replacing slower culture-based methods with rapid QPCR and other molecular methods. In the US, however, the standard protocol is based on assays of the more general group of fecal coliforms (of which 90%–95% are E. coli), which currently cannot be easily or accurately replicated via QPCR because primer-probe design would be compromised, given the large number of potential species to quantify within this taxonomically diverse group of bacteria. Thus, changing to rapid molecular techniques for shellfish health monitoring in the US would require both a shift to an E. coli standard and lengthy approval of a new molecular method.

Typically, local management agencies cannot fund the protocol adoption process, whereas academic researchers who have developed a new technique get little benefit from pushing it through the process. On the other side, the potential commercial market for new water quality testing protocols (on the order of US$100 million for shellfish [11] and US$15 million for recreational waters [12] annually in the United States) pales in comparison to other well-known incubations of lab-based technologies into commercial products, such as home pregnancy tests (US$229 million annually [13]), glucose test strips (US$2.5 billion [14]), and prescription drugs (US$200 billion [15]). So large, clinically based biotechnology corporations are not likely to take the risk in shepherding a long approval process because there is little commercial upside. Without this support, federal regulatory agencies need to step in to support adoption of molecular methods that can be used by resource management agencies.

Finally, as with all science to policy pathways, politics plays a role in adopting new water quality protocols. The Beach Protection Act of 2007 introduced by Representative Pallone from New Jersey—a state that has experienced increasing numbers of beach closures from 2005–2007—was written to clarify existing policy mandates to the EPA, which was working to advance rapid water quality testing methods. The bill adds the term “rapid” (defined as providing results within two hours) to the description of approved testing methods, and requires that results be posted “within 24 hours” rather than “promptly,” as currently required. The bill would also double the beach water quality testing budget from US$30 to US$60 million. Although the bill passed the US House of Representatives without significant opposition, in the Senate it was combined into an omnibus bill that did not pass. Such packaging of unrelated bills is routine, and illustrates how “politics” and “process” can kill policies based on sound science.

## Untangling the Molecules to Policy Pathway

What emerges from these examples is a tangled web of rapidly developing technologies, institutional needs and constraints, and the complex practice of policy making. We suggest that meaningful dialogues between scientists and resource managers can be a key first step in untangling this web into a coherent science to policy pathway. Indeed, the insights in this paper, as well as working collaborations between molecular scientists and managers, emerged from a “Molecules to Policy” working group we organized at the Duke University Marine Laboratory. Although the group focused on the Chesapeake Bay and North Carolina coastal region, its participants represented US state and federal resource agencies responsible for aquaculture, marine and anadromous fishery resources, sea turtle conservation, invasive species, and water quality monitoring for both recreational and shellfish harvesting waters. The wide range of management agencies, jurisdictions, and conservation problems represented makes both our general approach and many specific issues raised applicable to other efforts to incorporate new molecular techniques into management and environmental policy.

The workshop followed an interactive model that could easily be replicated in other places or for a different set of management issues. First, awareness about unresolved management questions was fostered by allowing managers to open the workshop by presenting their key challenges and outstanding research questions. Though molecular researchers had anticipated some questions raised by managers (“How do I determine the spatial and temporal boundaries of a given managed fisheries stock?”), other key questions (“How do we non-lethally determine sex in newly hatched turtles?”) led to unexpected discussions, because researchers assumed incorrectly that managers could sex turtles using standard non-molecular methods. This was followed by molecular researchers sharing relevant available and emerging molecular techniques. In combination, we identified key management questions in all of the managers' fields that might be addressed with new techniques (Table 1). This list of questions and techniques then became the nexus for identifying constraints to adoption of molecular techniques by guiding us to the following questions: “What technical issues need to be resolved?”; “Are there emerging non-molecular techniques that would do a better job?”; and “Are there institutional barriers that would hamper the implementation of this technique?”. Notably, this exercise had the additional benefit of highlighting opportunities for immediate collaborations, such as fisheries agencies sharing decades of archived genetic material and population estimate data with molecular researchers who will try to establish a relationship between effective (genetic) and actual (census) population size that might be used in future assessments of stock health.

**Table 1:**
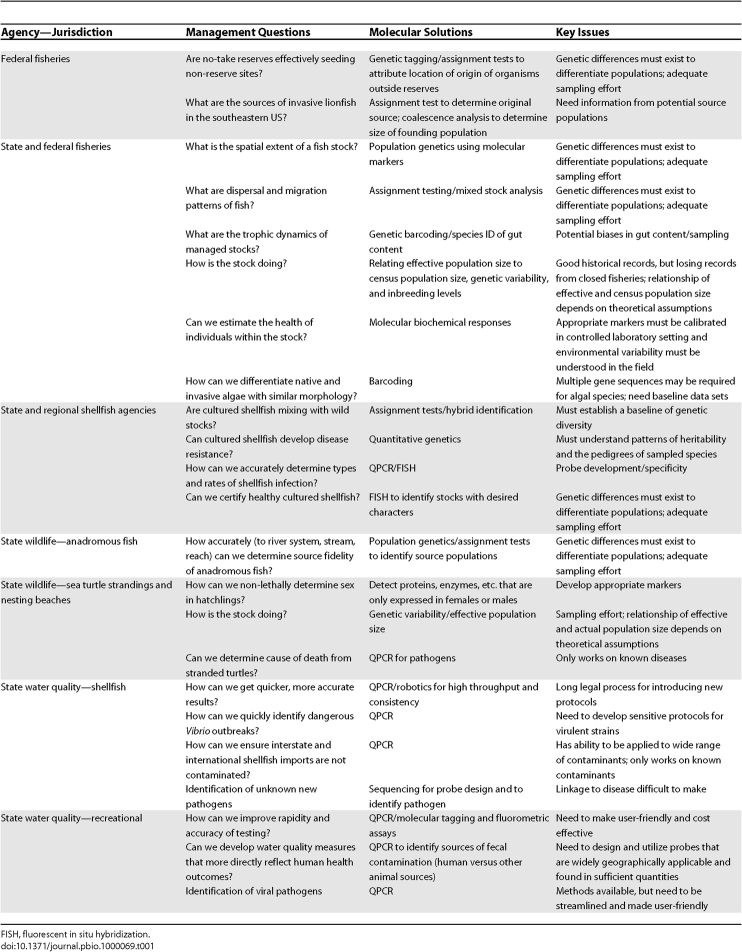
Selected Examples of Resource Management Questions That Can Be Addressed with Molecular Tools

Realizing the promise of incorporating molecular approaches in environmental policy depends foremost on a meaningful dialogue between scientists and resource managers to discover what tools may be beneficial to managers, followed by securing commitments from high-level governmental institutions to promote the adoption of these methods. Ultimately, the molecules to policy pathway illustrates the challenges of relating science to the needs of society. It must be driven by real world questions to be relevant, informed by the natural history of the study system to be accurate, and respectful of institutional norms and culture to be influential. Meeting these challenges will require mutual collaboration, a willingness to step outside of institutional comfort zones, and continual feedback between discoveries from the laboratory and the field and the practice of resource policy and management.

## Abbreviations

EPA: Environmental Protection Agency
ISSC: Interstate Shellfish Sanitation Conference
QPCR: quantitative polymerase chain reaction
SNP: single nucleotide polymorphism
